# Bone Protective Effects of Danggui Buxue Tang Alone and in Combination With Tamoxifen or Raloxifene *in vivo* and *in vitro*

**DOI:** 10.3389/fphar.2018.00779

**Published:** 2018-08-13

**Authors:** Li-Ping Zhou, Ka-Ying Wong, Hoi-Ting Yeung, Xiao-Li Dong, Hui-Hui Xiao, Amy G.-W. Gong, Karl W.-K. Tsim, Man-Sau Wong

**Affiliations:** ^1^Department of Applied Biology and Chemical Technology, The Hong Kong Polytechnic University, Kowloon, Hong Kong; ^2^Division of Life Science and Center for Chinese Medicine R&D, Hong Kong University of Science and Technology, Kowloon, Hong Kong; ^3^State Key Laboratory of Chinese Medicine and Molecular Pharmacology (Incubation), The Hong Kong Polytechnic University Shenzhen Research Institute, Shenzhen, China

**Keywords:** Danggui Buxue Tang, SERMs, postmenopausal osteoporosis, interactions, estrogen receptor

## Abstract

Danggui Buxue Tang (DBT), a traditional Chinese Medicine decoction containing *Astragali Radix* (AR) and *Angelicae Sinensis Radix* (ASR), is commonly prescribed for women in China as a remedy for menopausal symptoms. Previous study indicated that DBT stimulated cell growth and differentiation of human osteosarcoma MG-63 cells and exhibited estrogenic properties via estrogen receptors (ERs). The present study aimed to study the bone protective effects of DBT and its potential interactions with selective estrogen receptor modulators (SERMs, tamoxifen and raloxifene) in both *in vivo* and *in vitro* models as they act via similar ERs. Six-month-old Sprague-Dawley rats were randomly assigned to the following treatments for 12 weeks: (1) sham-operated control group with vehicle (sham), (2) ovariectomized group with vehicle (OVX), (3) OVX with 17β-estradiol (E2, 2.0 mg/kg day), (4) OVX with tamoxifen (Tamo, 1.0 mg/kg day), (5) OVX with raloxifene (Ralo, 3.0 mg/kg day), (6) OVX with DBT (DBT, 3.0 g/kg day), (7) OVX with DBT+Tamoxifen (DBT+Tamo), and (8) OVX with DBT+Raloxifene (DBT+Ralo). Effects of DBT and potential interactions between DBT and SERMs were also evaluated in MG-63 cells. DBT, tamoxifen, raloxifene, and their combinations significantly increased bone mineral density (BMD) and improved trabecular bone properties, including bone surface (BS), trabecular bone number (Tb.N), and trabecular bone separation (Tb.Sp), as well as restored changes in bone turnover biomarkers and mRNA expression of genes involved in bone metabolism in OVX rats. Furthermore, DBT, SERMs, and their combinations significantly increased serum estradiol and suppressed follicle stimulating hormone and luteinizing hormone in OVX rats, suggesting the possible involvement of the hypothalamus–pituitary–gonadal axis in mediating their bone protective effects. However, SERMs, but not DBT, significantly increased uterus index in OVX rats. DBT significantly induced ALP activity and estrogen response element-dependent transcription in MG-63 cells. Our study demonstrated that DBT alone and in combinations with SERMs could exert bone protective effects *in vitro* and *in vivo*.

## Introduction

Postmenopausal osteoporosis is a metabolic bone disease characterized by decreased bone mineral density (BMD) and increased bone fragility, resulting from the rapid drop of endogenous estrogen level that is experienced by 40% of postmenopausal women (Stein et al., 2014). Approximately 200 million postmenopausal women worldwide are affected and it causes over 2 billion cost annually (Parthan et al., 2013). Postmenopausal bone loss is the result of the reduced ovarian follicular activity and changes in female reproductive hormone levels [a decrease in estrogen and an increase in follicle stimulating hormone (FSH) and/or luteinizing hormone (LH) level] in the circulation of women during menopause (Lindsay, 2004). Indeed, hormone replacement therapy (HRT) was used to be the gold standard for management of postmenopausal osteoporosis (de Villiers et al., 2013) as it could significantly decrease the risk of bone fracture in postmenopausal women. However, with the evidence of the association of HRT use with an increased risk of breast cancer, endometrial cancer, and stroke in postmenopausal women, its clinical use for management of menopausal symptoms has been limited (Davison and Davis, 2003). Extensive efforts from the scientific communities are devoted to develop alternative regimen for management of menopausal symptoms and postmenopausal osteoporosis that is effective and without undesirable side effects.

Selective estrogen receptor modulators (SERMs) are estrogen receptors (ERs) ligands that serve as either full or partial agonists in some tissue, such as bone, but antagonists in other tissue, such as uterus and breast (Komm and Mirkin, 2014). Tamoxifen and raloxifene, two most widely used SERMs, have been shown to effectively reduce vertebral fracture in postmenopausal women (Nath and Sitruk-Ware, 2009). However, undesirable side effects in uterus have been reported in women prescribed with tamoxifen (Komm and Mirkin, 2014); while increase in vasomotor symptoms are associated with the use of SERMs in postmenopausal women (Taylor, 2009). These side effects might limit the long-term use of SERMs as a regimen for prevention and management of postmenopausal osteoporosis.

Phytoestrogens (plant-derived compounds with estrogenic properties) are the most popular alternative approach for management of postmenopausal health (Borrelli and Ernst, 2010). The most common examples of phytoestrogens are isoflavones, flavonoids, and lignans. Different types of phytoestrogens have been shown to attenuate bone loss associated with estrogen deficiency in both animal and human studies (Zhang et al., 2008; Bedell et al., 2014). In Asia, kidney-tonifying herbs are clinically used as alternatives to estrogen in alleviating menopause-related symptoms. Among them, Danggui Buxue Tang (DBT) is a simple Chinese herbal formula that is widely used by women in China to relieve menopausal symptoms such as hot flushes, sweating, anxiety, and mood swings (Wang et al., 2013). DBT is composed of *Astragali Radix* (AR) and *Angelicae Sinensis Radix* (ASR) at 5:1 ratio and was first described by Li Dongyuan in Neiwaishang Bianhuo Lun in AD 1247 (Zierau et al., 2014). Our laboratory has previously developed a standardized DBT extract (Dong et al., 2006) and demonstrated that the actions of DBT are in part mediated through ER-dependent mechanisms in mammary gland (Gao et al., 2007) and bone-derived cells (Choi et al., 2011) as well as ovariectomized Wistar rat model (Zierau et al., 2014). We have identified four bioactive components in these two herbs, including formononetin and calycosin from AR and ferulic acid and ligustilide from ASR (Dong et al., 2006). Pharmacokinetic study demonstrated that ferulic acid could enhance membrane permeabilities of calycosin and formononetin when AR and ASR were mixed at 5:1 ratio (Zheng et al., 2012), suggesting that the combination of the two herbs enhances the bioavailability of some of the phytoestrogens in the standardized DBT extract. Calycosin, an isoflavone, has been demonstrated to be the active component that predominantly trigger the estrogenic activities of DBT (Gong et al., 2016). Despite its well-known efficacy for alleviating menopausal symptoms, the efficacy of DBT for protecting against estrogen deficiency induced bone loss has not been systematically evaluated.

With the popularity of using herbal medicine as an alternative approach for management of menopausal symptoms, the safety of using those containing phytoestrogens among postmenopausal women is of utmost concern. As the effects of phytoestrogen are similar to those of estrogen and their effects are mediated through similar receptors and pathways, the use of phytoestrogen might carry similar risk-benefit profile as estrogen (Bedell et al., 2014). It is of particular concern to note that the number of breast cancer patients who seek to take supplements together with their standard treatment to prevent recurrence of breast cancer or bone loss as well as to manage their menopausal symptoms is increasing. Thus, it is of prime importance to determine if the combined use of SERMs with DBT will increase or decrease the estrogenic effects of SERMs at target tissues (such as bone) as well as non-target tissues (such as uterus).

The present study aimed to systematically evaluate the bone protective effects of DBT as well as to characterize the potential interactions between DBT and SERMs (tamoxifen and raloxifene) in mature ovariectomized rats as well as human osteosarcoma MG-63 cells.

## Materials and Methods

### Preparation and Chemical Analysis of Danggui Buxue Tang Extract

The extract of DBT decoction was prepared as previously described (Choi et al., 2011; Zierau et al., 2014). Briefly, fresh roots of 3-year-old *A. membranaceus var. mongholicus* and 2-year-old *A. sinensis* were purchased from Shanxi and Minxian of Gansu, China, respectively. 250 g of AR and 50 g of ASR were mixed exactly at the ratio of 5:1, boiled, and filtered. The residues were re-dissolved, boiled, dried under vacuum, and stored at -20°C. HPLC assay was performed for each herb to ensure their quality fulfill the requirement of the China Pharmacopoeia and/or Hong Kong Chinese Materia Medica Standard. The major chemical markers in AR are calycosin and formononetin while those in ARS are ferulic acid and ligustilide. The HPLC fingerprint and the constituents determined by LC–MS were used for quality control of DBT in the present study.

### Animal Experimental Design and Treatment

The present experiment was conducted under the animal license issued by Department of Health, Hong Kong Special Administrative Region Government, and the Hong Kong Polytechnic University Animal Subjects Ethics Sub-committee (Animal License No. 13-104; ASESC Case: 12/11). Seventy 6-month-old female Sprague-Dawley rats weighing 230 ± 20 g were purchased from The Chinese University of Hong Kong and housed in Centralized Animal Facilities (CAFs) of The Hong Kong Polytechnic University on a 12 h light/dark cycle. Water and food were available *ad libitum*. After 1 week of acclimatization, all the rats were given bilateral ovariectomy or sham operation. Upon recovery for 2 weeks, the OVX rats were randomly divided into seven treatment groups and orally administrated with vehicle, 17β-estradiol (2 mg/kg day) as positive control, tamoxifen (1 mg/kg day), raloxifene (3 mg/kg day), DBT (3 g/kg day), as well as combinations of DBT and either tamoxifen or raloxifene for 12 consecutive weeks. Dosages of drugs were conversed based on their clinical dosages and previous studies (Maricic and Gluck, 2002; Cooke et al., 2008; Ahmet-Camcioglu et al., 2009; Goss et al., 2009). Dosage of DBT was coverted from its clinical dose: 30 g of AR and 6 g of ASR (Zierau et al., 2014). During the whole recovery and treatment period, the rats were paired-fed with the phytoestrogen-free (AIN93-M) diets to remove the influences of phytoestrogen.

### Sample Collection

One day before sacrifice, each rat was housed individually in metabolic cage. 24 h urine was collected in metabolic cages, aliquoted, and stored at -80°C. Animals were sacrificed under anesthesia with ketamine/xylazine and blood was collected from rat abdominal aorta at sacrifice and aliquots of serum were stored at -80°C. Uteri were removed and weighed. The whole left leg and spine were collected and stored at -20°C. The right leg was freshly collected and stored at -80°C after removal of all soft tissues.

### Biochemical Assay of Serum and Urine Sample

Calcium (Ca) and phosphorus (P) level in serum and urine as well as urinary level of creatinine were measured by Arsenazo III UV method with an automatic analyzer HITACHI7100. The kits were purchased from Shanghai Kehua Bio-Engineering Co., LTD. (Shanghai, China). Urinary deoxypyridinoline (DPD) was determined by an enzyme immunoassay DPD EIA kit (QUIDEL Corporation, United States) and normalized by urinary creatinine. Serum level of osteocalcin (OCN), estradiol, LH, and FSH was measured by ELISA kit (Alfa Aesar), EIA kit (CayMan), and ELISA kit (CloudClone), respectively.

### BMD and Micro-CT Analysis

Bone properties of trabecular bone at proximal tibia and distal femur as well as lumbar vertebra were determined by Micro-CT (μCT40, Scanco Medical, Switzerland). The source energy selected for this study was 70 KVp and 114 μA with a resolution of 21 μm. Approximately 200 slices were done for each scan. The distal/proximal were defined as 4.2 and 2.2 mm away from femur/tibia head. Scanning was done at the metaphyseal area located 0.63 mm below the lowest point of the epiphyseal growth plate and extending 2.0 mm in the proximal direction. BMD (mg HA/ccm) and bone morphometric properties, including bone volume over total volume (BV/TV), trabecular bone number (Tb.N, mm^-1^), trabecular bone thickness (Tb.Th, mm), trabecular bone separation (Tb.Sp, mm), and bone surface (BS, mm^2^) were evaluated by contoured VOI images.

### Real-Time PCR Assay

Femoral head was cut off and homogenized in Trizol reagent by using Precellys 24 homogenizer (Bertin). Total RNA was extracted by following manufacturers’ instructions (Invitrogen, Carlsbad, CA, United States). Two micrograms of total RNA was reverse-transcribed into cDNA by using High-Capacity cDNA Reverse Transcription Kits (Applied Biosystems) at 25°C for 10 min, 37°C for 2 h, and 85°C for 5 min. One microliter of the cDNA product diluted by 10 times, 0.4 μl of forward and reverse primers (as shown in **Table 6**), 8.2 μl of DNase and RNase-free water, and 10 μl of SsoFast^TM^ EvaGreen^®^ Supermix (Bio-Rad) were mixed well to get the 20 μl of reaction system for real-time PCR. The iCycler with Iq5 Multicolor Real-Time PCR Detection System (Bio-Rad, IQ5) was used to perform and monitor the real-time quantitative PCR reaction. Conditions for each primer were optimized and the optimal condition was chosen for each primer (as shown in **Table 1**). For each gene, standard curve was established to determine the relative quantity of mRNA and the melting curve was used to assess the specificity of the amplification. Bone formation: (1) Alkaline phosphatase (*ALP*): specific product of osteoblast, indicating bone mineralization; (2) *OCN*: osteoblast-secreted non-collagenous protein. Bone resorption: (1) Interleukin-6 (*IL-6*) and (2) interleukin-1β (*IL-1β*): pro-osteoclastic cytokines, responsible for cartilage and bone destruction; (3) Receptor activator of nuclear factor kappa-B ligand (*RANKL*), and (4) osteoprotegerin (*OPG*): produced by osteoblast at different stages of maturity, indicating balance between osteoclast activity and osteoblast activity.

**Table 1:** Sequence of primers for bone remodeling-related genes.

Gene	sequence	Tm (°C)
GAPDH	CAAGTTCAACGGCACAGTCAAGG	60
	ACATACTCAGCACCAGCATCACC	
RANKL	GCAGCATCGCTCTGTTCCTGTA	61.4
	GCATGAGTCAGGTAGTGCTTCTGTG	
OPG	ACAATGAACAAGTGGCTGTGCTG	60
	CGGTTTCTGGGTCATAATGCAAG	
ALP	GTGGTGGACGGTGAACGGGAGAA	60.7
	ATGGACGCCGTGAAGCAGGTGAG	
OCN	GCAGCTTCAGCTTTGGCTACTCT	59.2
	CAACCGTTCCTCATCTGGACTTTA	
IL-1β	ATGAGAGCATCCAGCTTCAAATC	58.2
	CACACTAGCAGGTCGTCATCATC	
IL-6	CCAATTTCCAATGCTCTCCT	59
	ACCACAGTGAGGAATGTCCA	

### Cell Culture

Human osteosarcoma MG-63 cell (ATCC^®^ CRL-1427^TM^) was cultured in Minimum Essential Medium (MEM, Gibco) supplemented with 100 U/ml penicillin, 100 μg/ml streptomycin (Invitrogen, Carlsbad, CA, United States) and 10% fetal bovine serum (FBS, Gibco). The cultures were maintained in an incubator at 37°C in a humidified atmosphere of 95% O_2_ and 5% CO_2_.

### ALP Activity Assay

Cells were cultured with phenol red-free (PRF) medium containing 5% charcoal stripped fetal bovine serum (cs-FBS) before subjecting to treatment with vehicle, estradiol (10^-8^ M), tamoxifen (10^-12^–10^-6^ M), and raloxifene (10^-12^ to 10^-6^ M) as well as their combinations with DBT at 1 mg/ml (established in preliminary experiment) in PRF medium for 48 h. Upon treatment, 100 μl of passive lysis buffer (PLB) was added to each well to lyse cell. The ALP activity of cell lysate was measured by a LabAssay^TM^ ALP Kit (Wako, Japan) following manufacturer’s instruction. Total protein concentrations of the cell lysate were measured via Bradford method to normalize ALP activity.

### ERE Luciferase Activity Assay

Cells were transfected with 0.4 μg ERETkluc plasmid together with 0.01 μg of an inactive control plasmid pRL-TK, a Renilla luciferase control vector, by Lipofectamine^TM^ 2000 reagent (Invitrogen, Carlsbad, CA, United States) in PRF medium without antibiotics (Lau et al., 2009). 6 hours after transfection, the medium for transfection was discarded. Cells were subjected to vehicle, estradiol, (10^-8^ M), tamoxifen (10^-12^–10^-6^ M), and raloxifene (10^-12^–10^-6^ M) as well as their combinations with DBT at 1 mg/ml (established in preliminary experiment) in PRF medium for 24 h. Upon treatment, the medium was discarded and cells were lysed with 100 μl of PLB, collected for luciferase activity measurement. Luciferase activity was measured by a Dual Luciferase^®^ Reporter Assay System (Promega, E1960) and the signal detected by a TD-20/20 Luminometer (Turner Design, United States) following the manufacturer’s instructions. Results were expressed as ratio relative to control.

### Statistical Analysis

Data were reported as mean ± SEM. Inter-group differences were analyzed by one-way ANOVA with Tukey’s *post hoc* test. Interactions between drugs were determined by two-way ANOVA. *P*-value of <0.05 was considered statistically significant.

## Results

### Preparation and Standardization of DBT Extract

We defined the quality of DBT extract in the present study based on the content of the four chemical markers, calycosin and fomononetin from AR and ferulic acid and *Z*-linguizide from ASR, in the extract. As listed in **Table 2**, the contents of these markers in this batch od DBT extract were 809 μg of ferulic acid, 693 μg of calycosin, 165 μg of formononetin, and 212 μg of *Z*-ligustilide within 1 g of dried DBT extract, which were much higher than the one previously reported (Dong et al., 2006).

**Table 2:** Chemical constituents of DBT extract by LC–MS.

Marker name	Content (mg/g)	Minimum content (mg/g)
Ferulic acid	0.809	0.351
Calycosin	0.693	0.186
Formononetin	0.164	0.155
*Z*-Ligustilide	0.212	0.204

### DBT Alone Did Not Alter Body Weight, Uterus Weight, or Biochemical Parameters in OVX Rats

The effects of DBT and its potential interactions with SERMs on body weight, uterus index, and serum and urine chemistries were first evaluated in mature OVX rats in response to oral treatment for 12 consecutive weeks. As shown in **Table 3**, the OVX-induced body weight gain in rats was suppressed in response to treatment with estradiol, but not DBT, tamoxifen, nor raloxifene. Combination of DBT with tamoxifen, but not with raloxifene, also significantly reduced body weight gain in OVX rats. To assess the trophic effects on uterus, the ratio of uterus to body weight was determined as uterus index. Uterus index was significantly decreased in rats upon ovariectomy (**Table 3**, *p* < 0.001 vs. sham), suggesting that the surgery was successful. Estradiol, tamoxifen, and raloxifene significantly increased uterus index in OVX rats (**Table 3**, *p* < 0.001 vs. OVX). In contrast, DBT did not alter uterus index in OVX rats. Co-treatment of OVX rats with DBT and SERMs significantly increased uterus index in OVX rats (**Table 3**, *p* < 0.05 vs. OVX). Two-way ANOVA analysis indicated both tamoxifen and raloxifene interacted with DBT (**Table 3**, tamoxifen × DBT, *p* = 0.0011; raloxifene × DBT, *p* = 0.0032) for their effects on uterus weight. Serum Ca and P as well as urinary Ca and P excretion in rats were not altered by ovariectomy nor treatments with DBT, SERMs, and their combinations (**Table 3**).

**Table 3:** Effects of estradiol, tamoxifen, raloxifene, DBT, and their combinations on body weight, uterus index, biochemical parameters, and serum reproductive hormones in OVX rats.

Group	Change of body weight (% of the change)	Uterus index (mg/g)	Serum Ca (mg/L)	Serum P (mg/L)	Urinary Ca/Cr (mg/mg)	Urinary P/Cr (mg/mg)
Sham	2.36 ± 0.77	2.19 ± 0.09	101.2 ± 1.1	45.3 ± 2.6	0.10 ± 0.007	0.41 ± 0.04
OVX	8.15 ± 0.59	0.36 ± 0.03***	99.3 ± 0.7	47.2 ± 2.5	0.10 ± 0.009	0.41 ± 0.02
E2	-8.19 ± 0.81^∧∧∧^	1.56 ± 0.07^∧∧∧^	98.6 ± 0.8	45.6 ± 2.5	0.11 ± 0.007	0.42 ± 0.03
Tamo	2.25 ± 1.68	0.73 ± 0.05^∧∧∧^	99.5 ± 1.0	53.7 ± 1.8	0.10 ± 0.005	0.36 ± 0.04
Ralo	3.18 ± 1.88	0.70 ± 0.05^∧∧∧^	99.0 ± 0.7	53.6 ± 1.8	0.11 ± 0.010	0.42 ± 0.04
DBT	7.46 ± 0.99	0.50 ± 0.04	99.7 ± 1.0	49.5 ± 2.4	0.10 ± 0.009	0.39 ± 0.02
DBT+Tamo	0.85 ± 1.75^∧^	0.59 ± 0.03^∧^	98.9 ± 0.8	47.2 ± 1.3	0.10 ± 0.007	0.40 ± 0.02
DBT+Ralo	10.29 ± 1.90	0.61 ± 0.01^∧^	98.5 ± 0.8	49.8 ± 1.9	0.10 ± 0.008	0.42 ± 0.03
**Two-factor ANOVA *p*-value**						
Tamo	<0.0001	<0.0001	0.7613	0.3164	0.7455	0.5012
DBT	0.4582	1.0000	0.9402	0.3164	0.8207	0.5633
Tamo × DBT	0.7976	0.0011	0.6059	0.0406	0.6975	0.2869
Ralo	0.4716	<0.0001	0.3904	0.1287	0.3575	0.4552
DBT	0.0362	0.4896	0.9667	0.7439	0.9084	0.7877
Ralo × DBT	0.0123	0.0032	0.5882	0.1707	0.9774	0.8472

*^∗∗∗^*p* < 0.00‘ vs. sham; ^∧^*p* < 0.05, ^∧∧^*p* < 0.01, ^∧∧∧^*p* < 0.001 vs. OVX. Results are expressed as mean ± SEM, *n* = 7–9 rats per group. Ca, calcium; Cr, creatinine; P, phosphorus; Tamo, tamoxifen; Ralo, raloxifene.*

### DBT Alone and in Combination With SERMs Significantly Suppressed Bone Turnover Biomarkers in OVX Rats

**FIGURE 1:**
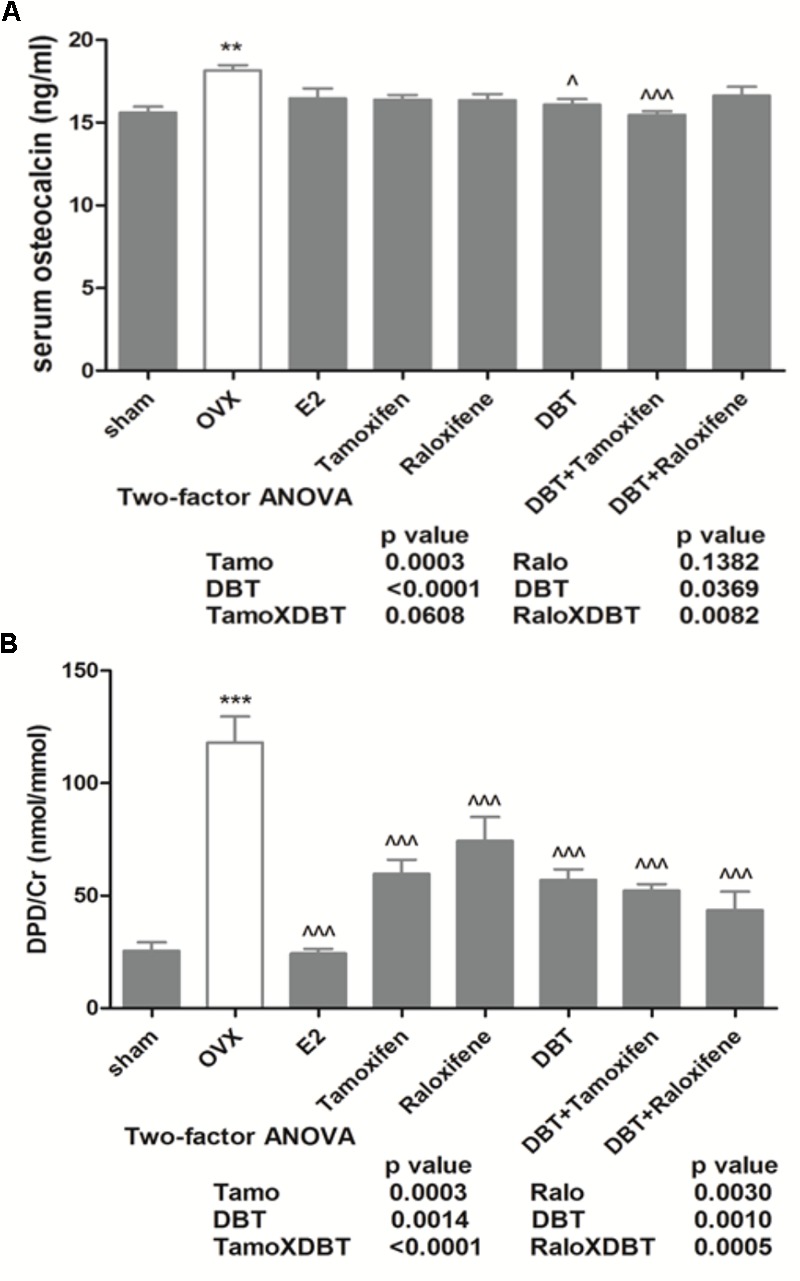
Effects of DBT, SERMs, and their combinations on serum level of OCN and urinary DPD of OVX rats. Six-month-old Sprague-Dawley rats were given ovariectomy or sham operation, After 2 weeks’ recovery, OVX rats were orally administrated with vehicle, 17β-estradiol (2.0 mg/kg day), tamoxifen (1.0 mg/kg day), raloxifene (3.0 mg/kg day), DBT (3 g/kg day), DBT+Tamoxifen, and DBT+Raloxifene for 12 consecutive weeks. **(A)** Serum level of OCN (ng/ml). **(B)** Urinary DPD (nmol/mmol). Data are expressed as mean ± SEM. ^∗∗^*p* < 0.01, ^∗∗∗^*p* < 0.001 vs. sham; ^∧^*p* < 0.05, ^∧∧∧^*p* < 0.001 vs. OVX. *n* = 7**–**9.

Serum OCN is a specific product of osteoblast and is regarded as a biomarker for bone formation (Nowacka-Cieciura et al., 2016) while urinary DPD is a breakdown product of collagen during bone resorption and is a biomarker for bone resorption (Pang et al., 2010). To assess the effects of DBT, SERMs, and their combinations on bone turnover, serum OCN and urinary DPD were measured. As shown in **Figure 1**, serum OCN and urinary DPD were significantly increased in OVX rats (*p* < 0.01 vs. sham) while treatment of OVX rats with 17β-estradiol significantly reduced the urinary level of DPD (*p* < 0.001 vs. OVX). Treatment with tamoxifen and raloxifene also significantly suppressed the increase in urinary DPD levels in OVX rats (*p* < 0.001 vs. OVX). Most importantly, treatment of OVX rats with DBT significantly suppressed OVX-induced increase in both serum OCN (**Figure 1A**, *p* < 0.05 vs. OVX) and urinary DPD (**Figure 1B**, *p* < 0.001 vs. OVX). Co-treatment of OVX rats with DBT and tamoxifen also markedly reduced both serum OCN and urinary DPD (*p* < 0.001 vs. OVX) while co-treatment of OVX rats with DBT and raloxifene significantly reduced urinary DPD level (*p* < 0.001 vs. OVX). Two-way ANOVA analysis suggested that DBT interacted with raloxifene for their effects on both serum OCN and urinary DPD (raloxifene × DBT, *p* = 0.0082 and *p* = 0.0005, respectively) while interacted with tamoxifen to alter urinary DPD in OVX rats (tamoxifen × DBT, *p* < 0.0001).

### DBT Alone and in Combination With SERMs Significantly Restored the Serum Level of Estradil, FSH, and LH in OVX Rats

**FIGURE 2:**
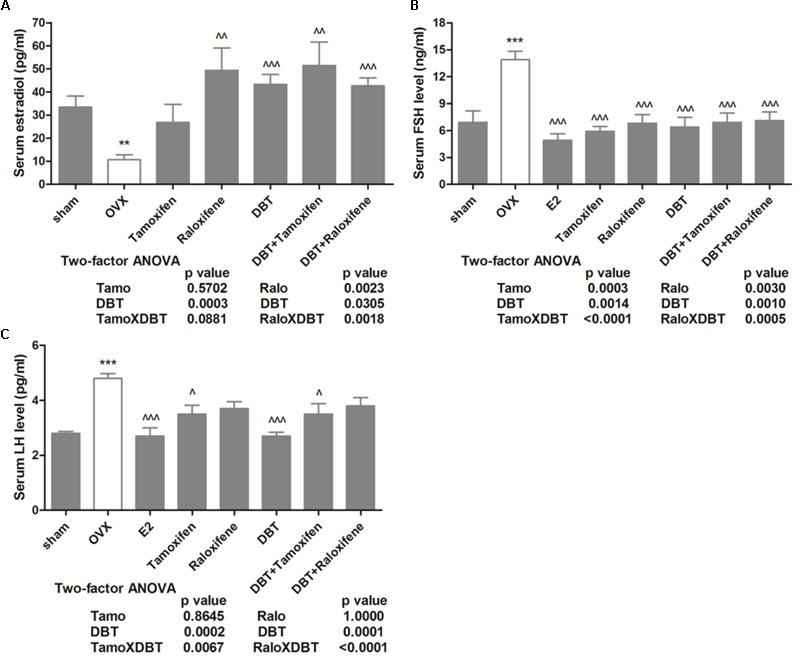
Effects of DBT, SERMs, and their combinations on serum level of estradiol, follicle-stimulating hormone, and LH of OVX rats. Serum level of estradiol, FSH, and LH were measured by EIA kit (CayMan) and ELISA kit (CloudClone), respectively. **(A)** Serum level of estradiol (pg/ml); **(B)** serum level of FSH (ng/ml); **(C)** serum level of LH (pg/ml). Data are expressed as mean ± SEM. ^∗∗^*p* < 0.01, ^∗∗∗^*p* < 0.001 vs. sham; ^∧^*p* < 0.05, ^∧∧^*p* < 0.01, ^∧∧∧^*p* < 0.001 vs. OVX. *n* = 7–9.

Both the decrease in estradiol level and the increase in FSH level are believed to be related to the development of postmenopausal osteoporosis and are accompanied by a sharp increase in the bone resorption biomarkers (Sowers et al., 2003; Sun et al., 2006). To determine changes in reproductive hormones in OVX rats upon treatments, serum levels of estradiol, FSH, and LH in rats were measured. Serum estradiol levels were significantly decreased in OVX rats, accompanied with the significant increase in levels of LH and FSH (**Figure 2**, *p* < 0.01 vs. sham). As expected, treatment of OVX rats with estradiol significantly increased serum estradiol and suppressed increase in FSH and LH levels (**Figure 2**, *p* < 0.001 vs. OVX). Serum estradiol increased in OVX rats upon treatment with raloxifene, DBT, as well as DBT in combination with SERMs (**Figure 2A**, *p* < 0.01 vs. OVX). Serum FSH levels were reduced in OVX rats in response to treatment with tamoxifen, raloxifene, DBT alone, and in combination with SERMs (**Figure 2B**, *p* < 0.001 vs. OVX). Serum LH levels in OVX rats were significantly reduced in response to tamoxifen, DBT, as well as their combination (**Figure 2C**, *p* < 0.05 vs. OVX). The levels of serum reproductive hormones in OVX rats treated with SERMs alone were not statistically different from those in OVX rats treated with respective SERMs in combination with DBT. Two-way ANOVA analysis indicated DBT and SERMs interacted to alter reproductive hormones (tamoxifen × DBT, FSH: *p* < 0.0001, LH: *p* = 0.0067; raloxifene × DBT, E2: *p* = 0.0018, FSH: *p* = 0.0005, LH: *p* < 0.0001) in OVX rats.

### DBT Alone and in Combination With SERMs Significantly Increased BMD and Improved Bone Properties in OVX Rats

**FIGURE 3:**
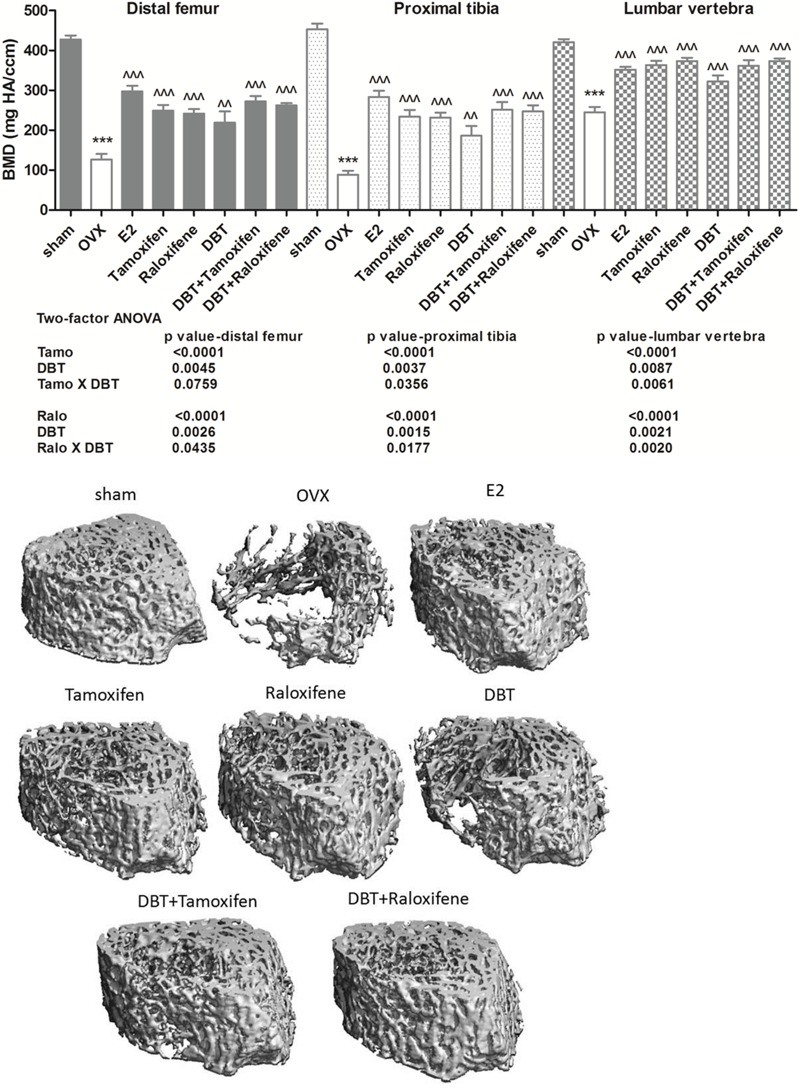
Effects of DBT, SERMs, and their combinations on bone micro-architecture (distal femur) and BMD of OVX rats. BMD of proximal tibia, distal femur, and lumbar vertebra were determined by micro-CT. Data are expressed as mean ± SEM. ^∗∗∗^*p* < 0.001 vs. sham; ^∧∧^*p* < 0.01, ^∧∧∧^*p* < 0.001 vs. OVX. *n* = 7–9.

**FIGURE 4:**
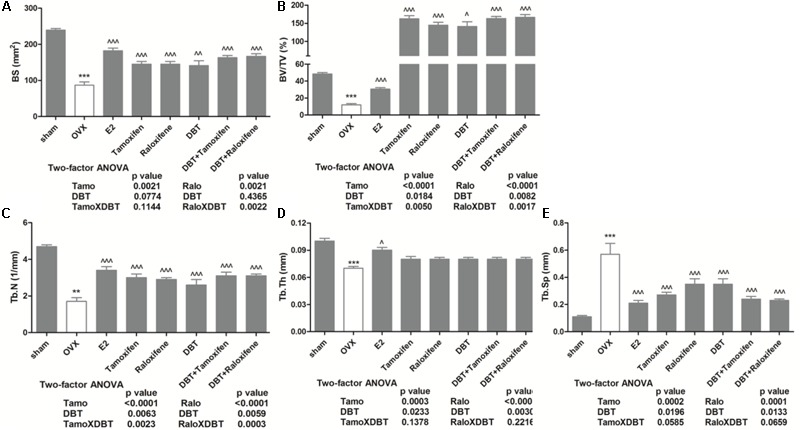
Effects of DBT, SERMs, and their combinations on bone properties (distal femur) of OVX rats. BMD of distal femur were determined by micro-CT. **(A)** Bone surface (mm^2^); **(B)** BV/TV (%); **(C)** trabecular bone number (1/mm); **(D)** trabecular bone thickness (mm); **(E)** trabecular bone separation (mm). Data are expressed as mean ± SEM. ^∗∗^*p* < 0.01, ^∗∗∗^*p* < 0.001 vs. sham; ^∧^*p* < 0.05, ^∧∧^*p* < 0.01, ^∧∧∧^*p* < 0.001 vs. OVX. *n* = 7–9.

The effects of DBT and its potential interactions with SERMs on trabecular bone properties in OVX rats were evaluated using micro-computed tomography (micro-CT). **Figure 3** clearly indicated that ovariectomy significantly reduced BMD at distal femur, proximal tibia, and lumbar vertebra in rats (*p* < 0.001 vs. sham). Treatment with estradiol, DBT, SERMs alone, and in combination with DBT significantly increased BMD in OVX rats (*p* < 0.01 vs. OVX). **Figure 4** and **Table 4** shows the effects of DBT, SERMs, and their combination on the bone micro-architectural properties at distal femur (**Figure 4**), proximal tibia, and lumbar vertebra (**Table 4**) in OVX rats. As expected, BS, bone volume/total volume (BV/TV), trabecular bone number (Tb.N), and trabecular thickness (Tb.Th) significantly decreased while trabecular separation (Tb.Sp) significantly increased in OVX rats at all the three bone sites (*p* < 0.01 vs. sham). Treatment of OVX rats with estradiol, tamoxifen, raloxifene, and DBT alone significantly increased BS, BV/TV, and Tb.N as well as decreased Tb.Sp of bone at all scanned sites (*p* < 0.05 vs. OVX). Treatment of OVX rats with DBT in combination with tamoxifen or raloxifene also significantly improved bone properties at these sites (*p* < 0.05 vs. OVX). BMD and bone properties at all three sites in OVX rats treated with SERMs alone were not statistically different from those in OVX rats treated with respective SERMs in combination with DBT. Two-way ANOVA analysis indicated that DBT interacted with tamoxifen to alter BMD at proximal tibia and lumbar vertebra and with raloxifene to alter BMD at all three sites in OVX rats (**Figure 3**, *p* < 0.05). Similarly, DBT interacted with both SERMs to alter bone microarchitectural properties at all three sites in OVX rats (**Table 4**, *p* < 0.05).

**Table 4:** Effects of DBT, tamoxifen, raloxifene, and their combinations on trabecular bone properties of OVX rats.

	Proximal tibia					Lumbar vertebra				
	BS (mm^2^)	BV/TV (%)	Tb.N (1/mm)	Tb.TH (mm)	Tb.Sp (mm)	BS (mm^2^)	BV/TV (%)	Tb.N (1/mm)	Tb.TH (mm)	Tb.Sp (mm)
Sham	228.8 ± 9.4	51.7 ± 2.3	5.0 ± 0.1	0.10 ± 0.004	0.10 ± 0.01	89.9 ± 2.7	44.2 ± 1.2	4.1 ± 0.1	0.11 ± 0.001	0.14 ± 0.01
OVX	55.0 ± 7.7**	7.6 ± 1.0***	1.2 ± 0.2***	0.06 ± 0.001***	0.86 ± 0.14***	62.6 ± 5.2**	20.9 ± 1.6***	2.6 ± 0.2***	0.08 ± 0.001***	0.31 ± 0.03***
E2	155.1 ± 10.3^∧∧^	25.5 ± 2.3^∧∧∧^	3.4 ± 0.2^∧∧∧^	0.08 ± 0.002^∧^	0.21 ± 0.01^∧∧∧^	90.4 ± 3.3^∧∧^	35.0 ± 1.0^∧∧∧^	3.7 ± 0.1^∧∧∧^	0.09 ± 0.002^∧∧^	0.18 ± 0.01^∧∧∧^
Tamo	128.5 ± 10.7^∧∧^	20.7 ± 2.1^∧∧^	2.9 ± 0.2^∧∧∧^	0.07 ± 0.003	0.29 ± 0.03^∧∧∧^	89.7 ± 5.9^∧^	36.6 ± 1.4^∧∧∧^	3.7 ± 0.1^∧∧∧^	0.10 ± 0.002^∧∧∧^	0.18 ± 0.01^∧∧∧^
Ralo	117.3 ± 6.1^∧∧^	19.9 ± 1.5^∧^	2.9 ± 0.1^∧∧∧^	0.07 ± 0.004	0.29 ± 0.02^∧∧∧^	93.0 ± 4.7^∧^	37.5 ± 1.1^∧∧∧^	3.7 ± 0.1^∧∧∧^	0.10 ± 0.002^∧∧∧^	0.17 ± 0.01^∧∧∧^
DBT	110.4 ± 18.6	17.2 ± 4.7	2.5 ± 0.3^∧∧∧^	0.07 ± 0.004	0.42 ± 0.06^∧∧∧^	81.3 ± 4.8^∧^	30.7 ± 1.9^∧∧^	3.4 ± 0.1^∧∧∧^	0.09 ± 0.004	0.21 ± 0.01^∧∧∧^
DBT+Tamo	129.4 ± 9.7^∧∧^	21.4 ± 2.5^∧∧^	3.1 ± 0.2^∧∧∧^	0.07 ± 0.003	0.26 ± 0.02^∧∧∧^	90.8 ± 5.6^∧∧^	36.1 ± 2.1^∧∧∧^	3.6 ± 0.1^∧∧∧^	0.01 ± 0.003^∧∧∧^	0.18 ± 0.01^∧∧∧^
DBT+Ralo	131.8 ± 10.1	22.4 ± 1.8^∧∧^	2.9 ± 0.2^∧∧∧^	0.077 ± 0.003^∧^	0.27 ± 0.02^∧∧∧^	81.4 ± 3.0^∧^	37.1 ± 1.0^∧∧∧^	3.5 ± 0.1^∧∧∧^	0.105 ± 0.002^∧∧∧^	0.18 ± 0.01^∧∧∧^
**Two-factor ANOVA *p*-value**										
Tamo	0.0010	0.0059	<0.0001	0.0364	<0.0001	0.0021	<0.0001	<0.0001	0.0364	<0.0001
DBT	0.0329	0.0871	0.0041	0.1394	0.0070	0.0774	0.0138	0.0041	0.1394	0.0070
DBT × Tamo	0.0385	0.1367	0.0296	0.2467	0.0171	0.1144	0.0070	0.0296	0.2467	0.0171
Ralo	0.0014	0.0028	<0.0001	0.0043	0.0001	0.0021	<0.0001	<0.0001	0.0043	0.0001
DBT	0.0063	0.0316	0.0038	0.0247	0.0087	0.4365	0.0030	0.0038	0.0247	0.0087
DBT × Ralo	0.0951	0.1949	0.0078	0.5882	0.0124	0.0022	0.0015	0.0078	0.5882	0.0124

*^∗∗^*p* < 0.01, ^∗∗∗^*p* < 0.00‘ vs. sham; ^∧^*p* < 0.05, ^∧∧^*p* < 0.01, ^∧∧∧^*p* < 0.001 vs. OVX. Results are expressed as mean ± SEM, *n* = 7–9 rats per group. BS, bone surface; Tb.N, trabecular bone number; Tb.Th, trabecular bone thickness; Tb.Sp, trabecular bone separation; Tamo, tamoxifen; Ralo, raloxifene.*

### DBT Alone and in Combination With SERMs Regulated the mRNA Expression of Genes Involved in Bone Metabolism in Femoral Head of OVX Rats

**FIGURE 5:**
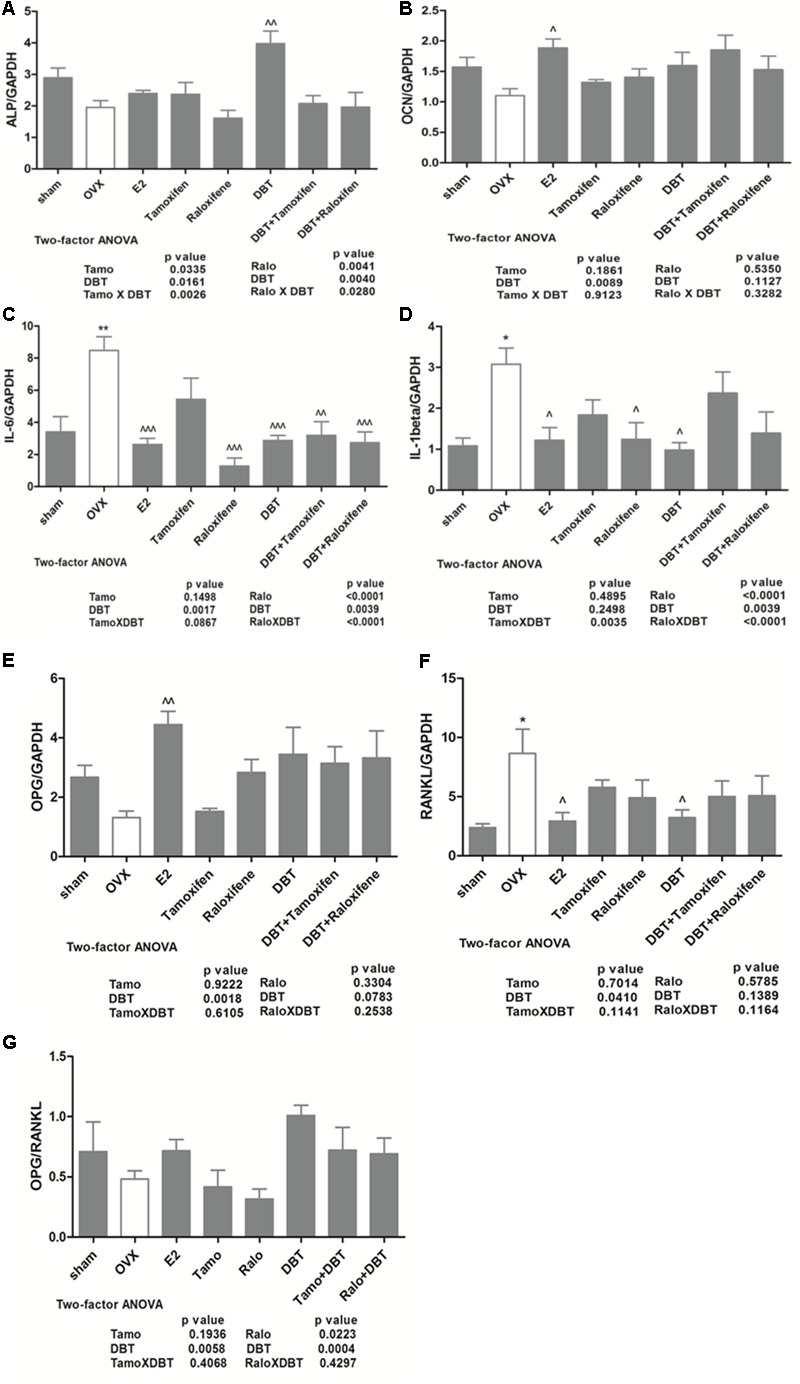
Effects of DBT, SERMs, and their combinations on mRNA expression of bone remodeling-related genes in femoral head of OVX rats. mRNA expression of bone remodeling-related genes in femoral head was determined by real-time PCR. **(A)** ALP mRNA expression; **(B)** OCN mRNA expression; **(C)** IL-6 mRNA expression; **(D)** IL-1β mRNA expression; **(E)** RANKL mRNA expression; **(F)** OPG mRNA expression; **(G)** OPG/RANKL ratio. Data are expressed as mean ± SEM. ^∗^*p* < 0.05, ^∗∗^*p* < 0.01 vs. sham; ^∧^*p* < 0.05, ^∧∧^*p* < 0.01 vs. OVX. *n* = 7–9.

Both ALP and OCN are products of osteoblast and well-validated markers for bone formation activity (Callaci et al., 2009). To determine the effects of DBT and its potential interaction with SERMs on bone formation, the mRNA expression of *ALP* and *OCN* in femoral head of rats were measured. The expression of *ALP* and *OCN* mRNA was slightly reduced in OVX rats when compared to sham rats, but the changes did not reach statistical significance (**Figures 5A,B**). Treatment of OVX rats with estradiol significantly increased *OCN* mRNA expression (*p* < 0.05 vs. OVX); while treatment with DBT significantly induced ALP mRNA expression (*p* < 0.01 vs. OVX). Interleukin-6 (IL-6) and IL-1β belong to inflammatory cytokines and stimulate bone resorption activity by regulating osteoproegrin (OPG) and RANKL system. Indeed, the OPG–RANKL system is vital to osteoclast differentiation and resorption activity (Callaci et al., 2009) in which OPG acts as decoy receptor and blocks the interactions between RANKL and its receptor RANK, therefore, attenuates its function in osteoclastogenesis. To determine the effects of DBT and its potential interaction with SERMs on bone resorption, the mRNA expression of *IL-6, IL-1ß, OPG*, and *RANKL* in femoral head of rats was measured. The expression of *IL-6, IL-1ß*, and RANKL mRNA was significantly higher while *OPG* mRNA expression was lower in OVX rats than those in sham (**Figures 5C–F**, *p* < 0.05 vs. sham). The ratio of *OPG*/*RANKL*, which is commonly used as an indicator for the control of osteoclastogenesis, decreased in OVX rats when compared to Sham group (**Figure 5G**). Treatment of OVX rats with estradiol significantly reduced *IL-6, IL-1ß*, and *RANKL* mRNA expression, promoted *OPG* mRNA expression, and the ratio of *OPG*–*RANKL* in femoral head (*p* < 0.05 vs. OVX). Raloxifene, but not tamoxifen, significantly reduced IL-6 (*p* < 0.001 vs. OVX) and IL-1β (*p* < 0.05 vs. OVX) mRNA expression in femoral head of OVX rats. Most importantly, treatment of OVX rats with DBT alone significantly reduced *IL-6* (*p* < 0.001 vs. OVX), *IL-1ß* (*p* < 0.05 vs. OVX), as well as *RANKL* mRNA expression and promoted the *OPG* mRNA expression as well as ratio of *OPG*–*RANKL*. Our results also showed that DBT in combination with tamoxifen and raloxifene significantly reduced *IL-6* mRNA expression in femoral head of OVX rats (*p* < 0.01 vs. OVX). mRNA expression of genes involved in bone metabolism in femoral head in OVX rats treated with SERMs alone was not statistically different from those in OVX rats treated with respective SERMs in combination with DBT. Two-way ANOVA suggested that tamoxifen interacted with DBT on regulation of mRNA expression of *ALP* (**Figure 5A**, *p* = 0.0026) and *IL-1ß* (**Figure 5D**, *p* = 0.0035) in femoral head of OVX rats. Raloxifene also interacted with DBT on regulation of these three genes *ALP* (**Figure 5A**, *p* = 0.0280), *IL-6* (**Figure 5C**, *p* < 0.0001), and additional *IL-1ß* (**Figure 5D**, *p* < 0.0001) in femoral head of OVX rats.

### DBT Significantly Increased ALP Activity and ERE Luciferase Activity in Human Osteosarcoma MG-63 Cells

**FIGURE 6:**
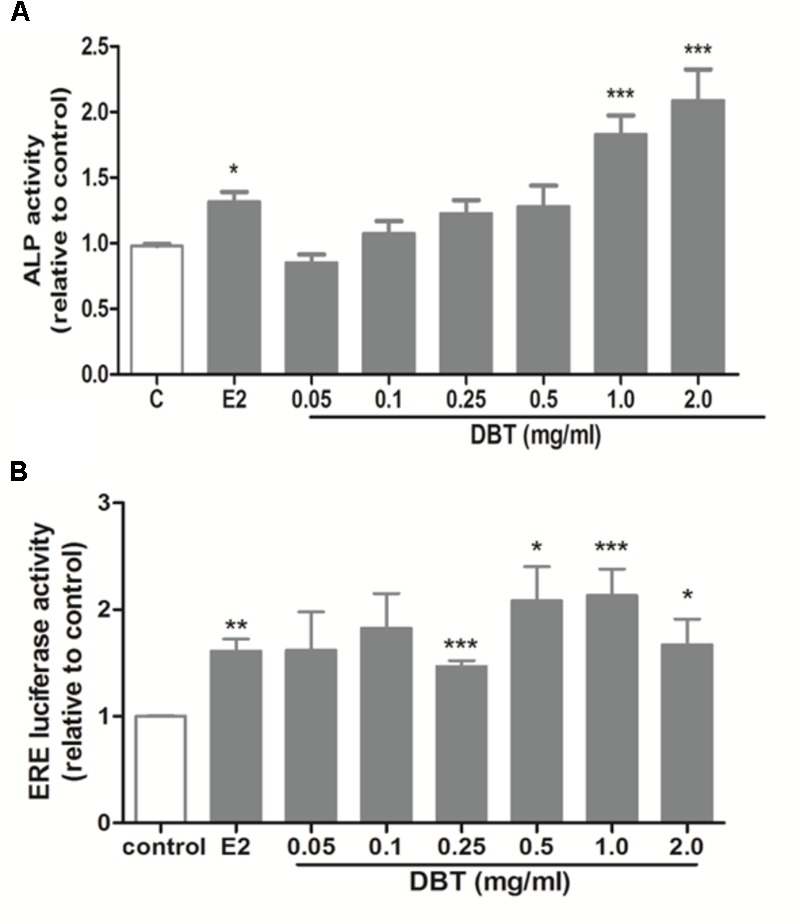
Effects of DBT on ALP activities and ERE luciferase activities in MG-63 cells. Human osteosarcoma MG-63 cells were treated with DBT at 0.05, 0.1, 0.25, 0.5, 1.0, and 2.0 mg/ml in PRF DMEM containing 5% cs-FBS for 48 h. **(A)** Dose-dependent effects of DBT on ALP activity. ALP activity of the cell lysate was measured by commercial kit and normalized by the total protein concentrations. **(B)** Dose-dependent effects of DBT on ERE luciferase activity in MG-63 cells transfected with ERETkluc plasmid together with an inactive control plasmid pRL-TK for 6 h and treated with DBT at 0.05, 0.1, 0.25, 0.5, 1.0, and 2.0 mg/ml. Luciferase activity was measured by a Dual Luciferase Receptor Assay System. Data are expressed as mean ± SEM. Results were from two independent experiments. ^∗^*p* < 0.05, ^∗∗^*p* < 0.01, ^∗∗∗^*p* < 0.001 vs. control; ^∧^*p* < 0.05, ^∧∧^*p* < 0.01, ^∧∧∧^*p* < 0.001 vs. tamoxifen or raloxifene alone. *n* = 3.

**FIGURE 7:**
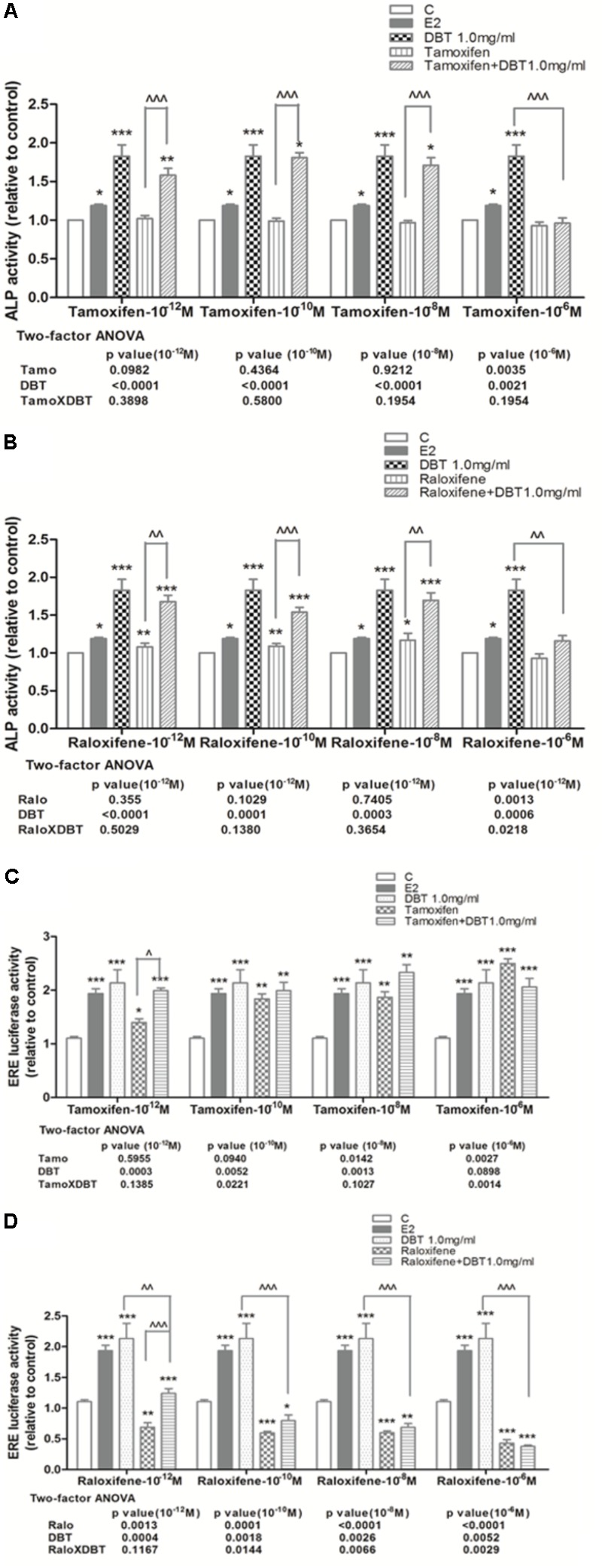
Effects of SERMs and their combinations with DBT on ALP activity and ERE luciferase activity of MG-63 cells. Human osteosarcoma MG-63 cells treated with DBT at 1.0 mg/ml were co-treated with different concentration (10^-12^–10^-6^ M) of tamoxifen and raloxifene in PRF DMEM containing 5% cs-FBS for 48 h. **(A)** Dose-dependent effects of tamoxifen alone and in combination with DBT on ALP activity; **(B)** dose-dependent effects of raloxifene alone and in combination with DBT on ALP activity; **(C)** dose-dependent effects of tamoxifen alone and in combination with DBT on ERE luciferase activity; **(D)** dose-dependent effects of raloxifene alone and in combination with DBT on ERE luciferase activity. Data are expressed as mean ± SEM. Results were from two independent experiments. ^∗^*p* < 0.05, ^∗∗^*p* < 0.01, ^∗∗∗^*p* < 0.001 vs. control; ^∧^*p* < 0.05, ^∧∧^*p* < 0.01, ^∧∧∧^*p* < 0.001 vs. tamoxifen or raloxifene alone. *n* = 3. Interactions between SERMs and DBT were analyzed by two-factor ANOVA.

The effects of DBT and its potential interactions with SERMs *in vitro* were studied using MG-63 cells. As shown in **Figure 6A**, DBT mimicked estradiol and significantly increased ALP activities in dose-dependent manner. In particular, DBT at 1 and 2 mg/ml increased ALP activity of MG-63 cell by 82 and 109%, respectively (*p* < 0.001 vs. control). To determine whether DBT exert estrogen-like responses via the activation of estrogen response element-dependent transcription, MG-63 cells were transfected with ERE luciferase reporter and subjected to treatment with different dosages of DBT. As shown in **Figure 6B**, DBT (0.05–2.0 mg/ml) significantly induced ERE-dependent luciferase activities in MG-63 cell in a way similar to estradiol (*p* < 0.05 vs. control). DBT at 1 mg/ml exerted the greatest stimulatory effect on ERE luciferase activity (*p* < 0.001 vs. control). To determine whether DBT interact with the actions of SERMs on ALP activities (**Figures 7A,B**) and ERE luciferase activities (**Figures 7C,D**), MG-63 cells were treated with different concentrations of tamoxifen or raloxifene (10^-12^–10^-6^ M) in the presence or absence of DBT at 1 mg/ml. Raloxifene (10^-12^–10^-8^ M, **Figure 7B**), but not tamoxifen (**Figure 7A**), significantly induced ALP activities in MG-63 cells (*p* < 0.05 vs. control). Upon co-treatment for 48 h, DBT at 1.0 mg/ml together with tamoxifen (*p* < 0.01 vs. tamoxifen alone) and raloxifene (*p* < 0.01 vs. raloxifene alone) at lower concentrations (10^-12^–10^-8^ M) significantly increased ALP activities, while SERMs at 10^-6^ M blunted the stimulatory effects of DBT on ALP activities (*p* < 0.001 vs. DBT alone). In contrast, the responses of ERE luciferase activities in MG-63 cells to treatment with tamoxifen, raloxifene, and DBT were different. Tamoxifen significantly increased ERE luciferase activities in MG-63 cells in a dose-dependent manner (**Figure 7C**, *p* < 0.05 vs. control) while raloxifene inhibited ERE luciferase activities in MG-63 cells at all the treated dosages (**Figure 7D**, *p* < 0.01 vs. control). DBT at 1 mg/ml significantly enhanced the stimulatory effects of tamoxifen at 10^-12^ M but not the other concentration (**Figure 7C**, *p* < 0.01 vs. tamoxifen alone), and could only reverse the inhibitory effects of raloxifene at 10^-12^ M. Two-way ANOVA analysis indicated that DBT interacted with raloxifene to alter ALP activities (raloxifene × DBT: *p* = 0.0218 at 10^-6^ M) and interacted with both tamoxifen and raloxifene to alter ERE luciferase activities (*p* < 0.05, tamoxifen at 10^-10^ M and 10^-6^ M; *p* < 0.05, raloxifene at 10^-10^ M, 10^-8^ M, and 10^-6^ M) in MG-63 cells.

## Discussion

Danggui Buxue Tang decoction has been traditionally believed to raise “Qi” (vital energy) and nourish the “Blood” (body circulation) and has been clinically prescribed for postmenopausal women to improve health and alleviate menopause symptom in Asia and China. *AR* and *Angelica Sinensis Radix*, two herbs contained in DBT, are commonly used for treatment of age-related diseases and have been demonstrated to stimulate bone cell proliferation, increase bone formation, and reduce bone resorption in patients (Choi et al., 2011). The present study was the first to demonstrate that DBT at its clinical dosage protected against estrogen deficiency-induced bone loss in OVX rats, possibly via inhibiting bone turnover and modulating the regulation of hypothalamus–pituitary–gonadal (HPG) axis without inducing uterotrophic effects. In addition, DBT in combination with either tamoxifen or raloxifene could also effectively protect against estrogen-induced bone loss in OVX rats. Two-way ANOVA analysis indicated that DBT interact with tamoxifen and raloxifene in altering uterus weight in OVX rats.

Danggui Buxue Tang at 3 g/kg day is effective in protecting against bone loss associated with estrogen deficiency. This dose equals to 33.33 g AR and ASR for human weighing 70 kg, which is in close proximity to the clinical dosages of DBT, 30 g (10 qian) of AR, and 6 g (2 qian) of ASR, described by Li Dongyuan in *Neiwaishang Bianhuo Lun* (Zierau et al., 2014). Importantly, our study showed that DBT attenuated bone loss associated with estrogen deficiency to comparable extent to that of SERMs at their clinical dosages. The dosages of tamoxifen and raloxifene used in the present study are equivalent to 11 and 33.3 mg/kg day in human, respectively, which are close to the clinical dosages of them (10–40 mg/kg day of tamoxifen for treatment of breast cancer, 60 mg/kg day of raloxifene for treatment and prevention of osteoporosis as well as prevention for breast cancer) (Maricic and Gluck, 2002; Cooke et al., 2008). Indeed, our study was the first to compare the bone protective activities between SERMs and DBT and found that these agents at their clinical dosages exerted similar bone protective effects in OVX rats.

Trabecular bone comprises only 20% of the whole bone, but trabecular bone loss is more rapid than cortical bone especially during the first 10 years after menopause (Zebaze et al., 2010). For rats, the trabecular bone loss begins in the metaphysis regions of the long bone and spine (Recker et al., 2011). Thus, the protective effects of DBT on bone mass of OVX rats were evaluated at metaphysis of tibia, femur, and lumber spine by micro-CT. Our results revealed that DBT could increase BMD at both long bone and spine. In addition to BMD, bone microarchitecture and bone properties were also measured. Bone properties, like BV/TV, Tb.N, Tb.Th, and Tb.Sp, at distal femur, proximal tibia, and lumbar spine were improved in OVX rats upon treatment with DBT at its clinical dose. The osteoprotective effects of DBT appear not to be site-specific as it could significantly improve bone properties at all sites measured. In contrast, raloxifene, as the only SERM on market for reducing bone fracture, decreases the incidence of vertebral fracture by 30–50% in postmenopausal women with osteoporosis but has not been shown to be effective in prevention of non-vertebral fracture (Ettinger et al., 1999). Although clinical use of tamoxifen for prevention of bone fracture is somewhat controversial, it was shown to be effective in preventing fracture at both vertebral and non-vertebral sites, especially hip fracture, in large population-based studies (Cooke et al., 2008; Tzeng et al., 2015). The present study confirmed the preferential protective effects of raloxifene in vertebra and demonstrated the efficacy of tamoxifen at both vertebral and non-vertebral sites in OVX rats. Co-treatment of SERMs with DBT appeared to provide some additional benefits to their protective effects on BMD and bone properties (like Tb.N and Tb.Sp) in long bone, but the improvement did not reach statistical significance.

The bone protective effects of DBT in OVX rats appeared to be mediated by suppressing bone turnover. Our results clearly showed that 17β-estradiol, tamoxifen, and raloxifene significantly inhibited the levels of urinary DPD in OVX rats as reported by others previously (Canpolat et al., 2010). Moreover, DBT significantly suppressed the levels of serum OCN and urinary DPD in OVX rats in which the extent of inhibiton was 57 and 11% for DPD and OCN, respectively. At the transcriptional level, DBT significantly altered mRNA expression of genes involved in bone formation and bone resorption in femoral head of OVX rats. *ALP* and *OCN*, genes involved in bone formation, are expressed during osteoblastic differentiation. Indeed, the mRNA expression of *ALP* and *OCN* was not significantly altered by ovariectomy in rat bone in our study. ALP has been demonstrated to be present in a large number of cells including preosteoblast while OCN present in relatively mature osteoblast (Weinreb et al., 1990). Their mRNA expression in bone from OVX rats was significantly increased only by treatment with DBT and 17β–estradiol, respsectively, suggesting that DBT might regulate the earlier stage of osteoblast differentiation while 17β–estradiol regulate the later stage. In contrast, mRNA expression of *IL-1ß, IL-6*, and *RANKL*, genes involved in bone resorption, was significantly induced in femoral head by ovariectomy in rats. As expected, treatment of OVX rats with 17β–estradiol suppressed the mRNA expression of gene involved in bone resorption and induced *OPG* mRNA expression in femoral head, indicating their inhibitory actions on the process of osteoclastogenesis. Treatment of OVX rats with DBT and raloxifene, but not tamoxifen, signifiantly suppressed the mRNA expression of *IL-6* and *IL-1ß* in femoral head. Moreover, DBT could significantly suppress *RANKL* mRNA expression in femoral head of OVX rats, indicating its potential role in suppressing osteoclastogenesis. Thus, both the results of bone gene expression and bone turnover markers in the present study suggest DBT could suppress bone turnover in OVX rats and its actions appear to be different from those of estradiol, tamoxifen, and raloxifene. Moreover, its preferential inhibitory effects on urinary DPD levels and mRNA expression of bone resorption genes indicate that its bone protective effects might be mediated by suppessing the process of bone resorption.

The systemic actions of DBT might be mediated via its actions on the HPG axis. Estrogen deficiency has long been thought to be the sole reason for bone loss and regarded as the primary target of treatment in both genders. However, estrogen deficiency itself does not fully explain for the bone loss in hypogonadism conditions (Yeh et al., 1996, 1997). In particular, FSH was found to directly regulate bone mass by stimulating osteoclastogenesis and bone resorption and its circulating levels were associated with the changes in bone turnover biomarkers in postmenopausal women (Garcia-Martin et al., 2012). The increase in circulating FSH is found to begin even before the decrease in estrogen and this increase in FSH is accompanied with a boost in bone markers. Such correlation between the circulating levels of FSH and bone markers, especially bone resorption marker urinary DPD, was also observed in the present study. Our results showed that treatment of OVX rats with 17-b estradiol and DBT significantly suppressed ovariectomy-induced serum FSH and LH levels and induced serum estradiol levels in OVX rats upon treatment for 12 weeks. The actions of SERMs on these hormones in OVX rats appear to be slightly different. Tamoxifen significantly suppressed serum FSH and LH levels but did not induce serum estradiol levels in OVX rats; whereas raloxifene significantly suppressed serum FSH and induced serum estradiol levels in OVX rats. As both FSH and estradiol play essential role in controlling bone mass, our results suggest that the *in vivo* bone protective actions of DBT as well as SERMs might be mediated via their effects on modulating the secretion of these hormones by the HPG axis.

Our results showed that DBT did not alter the uterus weight in OVX rats despite its stimulatory effects on circulating estrogen level. Uterus is one of the sensitive target tissues of estrogen and also the tissue that the side effects of estrogen and SERMs are frequently reported (Goss et al., 2007). Indeed, our study clearly showed that tamoxifen, raloxifene, and 17β–estradiol significantly increased uterus index in OVX rats. The fact that DBT increased serum estradiol without inducing uterus weight suggested that DBT might alter the responses of uterus to circulating estrogen in OVX rats. However, the underlying mechanism for the differential actions of DBT in uterus remains unclear. Previous studies reported that phytoestrogens could facilitate the clearance of estrogens from local tissues, such as uterus and breast, and catabolize the estrogens to more benign 2-hydroxylated metabolites (Wood et al., 2007). Thus, it is also possible that DBT might alter the clearance of estrogens in reproductive tissues, thereby reducing the potential side effects in these tissues. Further study will be needed to investigate the actions of DBT on tissue sensitivity to estradiol as well as local estradiol metabolism.

Esstrogen receptors α and β are expressed in bone that mediate the direct effects of estrogen, resulting in decreased bone resorption and formation activity (Khalid and Krum, 2016). Phytoestrogens, as alternatives to estrogen, also exert estrogen-like activities possibly via their direct but weak affinity for ERs. Indeed, three major constitutents in DBT, calycosin, and fomonenotin from AR and ferulic acid from ASR, are flavonone phytoestrogens. It would be a concern for postmenopausal women who are routinely prescribed with SERMs (such as tamoxifen and raloxifene) to consume herbal products containing phytoestrogens (such as DBT) for management of menopausal symptoms as the latter might interfere with the efficacy of the prescription drugs. The present study using OVX rats demonstrated that co-treatment with DBT at its clinical dose did not alter the efficacy of SERMs in bone nor worsen their side effects in the uterus. Moreover, DBT appeared to exert its beneficial effects on bone also via the modulation of hormonal systems in OVX rats. Thus, it is possible that the level of major phytoestrogens in DBT, upon metabolic activation and clearance, will not be high enough to interfere with binding activities of SERMs toward ERs in different tissues. Future study will be needed to characterize the tissue distribution of major phytoestrogens in OVX rats upon long-term treatment with DBT.

The *in vitro* studies demonstrated the differences between the actions of tamoxifen and raloxifene in bone cells. Tamoxifen significantly induced but raloxifene inhibited ERE-dependent luciferase activities in human osteosarcoma MG-63 cells, indicating that tamoxifen might act as agonist of ERs while raloxifene as antagonist in osteoblast MG-63 cells. Although correlation exists between ERE-dependent transcriptional activity and cell differentiation, their effects on ALP activities in osteoblast might also be mediated by ERE-independent transcriptional events. Our results indicated the possible mechanistic differences between tamoxifen and raloxifene and that the osteoprotective effects of them might be mediated via both ERE-dependent and ERE-independent mechanisms. In addition, our study showed that the potent stimulatory effects of DBT on ALP activity and ERE-dependent transcriptional activity were offseted by co-treatment with tamoxifen and raloxifene. Thus, the *in vitro* study supported our speculation that the effects of phytoestrogens present in DBT and SERMs in estrogen-sensitive cells would be altered when they are co-adminstrated as their actions are mediated by similar ERs. However, such strong antagonizing effects of SERMs on the estrogenic actions of DBT in bone cells were not reflected from the results of our *in vivo* study. Although two-way ANOVA analysis indicated that interactions exist between SERMs and DBT on bone properties, co-administration of SERMs and DBT did not result in reduced beneficial effects of either treatment on bone.

**FIGURE 8:**
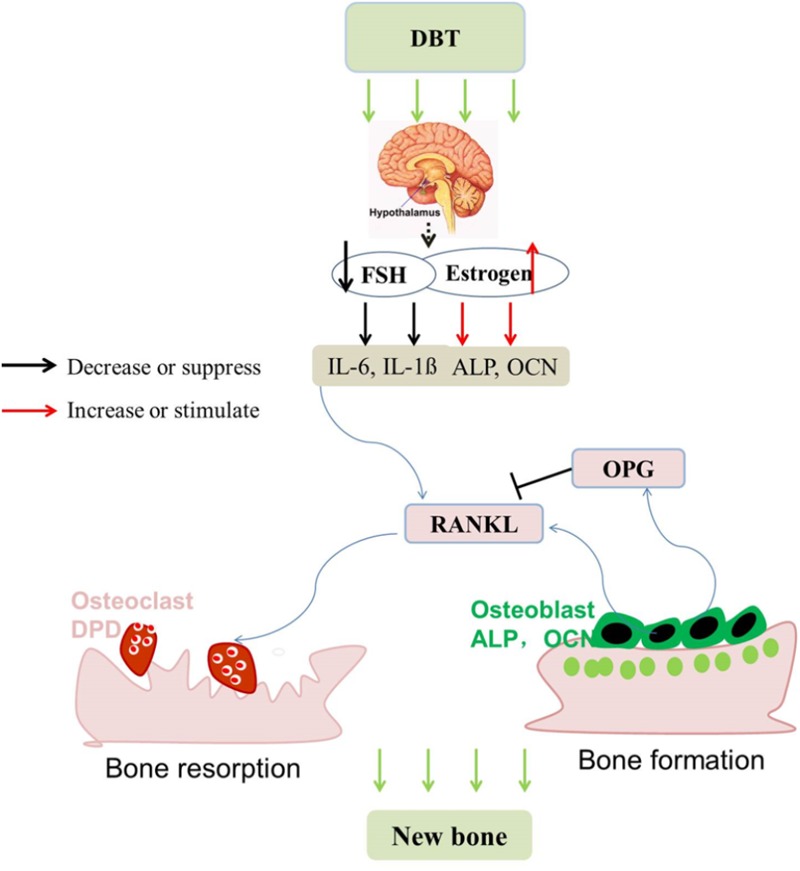
Possible mechanism for the anti-osteoporotic effects of DBT, SERMs, and their combinations in OVX rats. (1) DBT modulates the hypothalamus–pituitary–gonadal axis in OVX rats; (2) estradiol and FSH (and/or LH) regulate bone remodeling-related genes, mainly suppressing bone resorption and resulting in the bone protective activities.

Taken together, DBT at clinically relevant dose was effective in protecting against estrogen defiency induced bone loss without inducing uterus weight in OVX rats. Its bone protective effects appeared to be mediated by its actions in modulating the hypothalamus–pituitary–gonadal axis which restore the change in levels of estradiol, FSH, and LH; and subsequently regulated bone remodeling by primarily suppressing the bone resorption process (**Figure 8**). The bone protective effects of DBT and SERMs *in vivo* and in osteoblastic cells were not additive and these agents appeared to interact with each other in exerting bone protective effects *in vivo*. Future study will be needed to verify the clinical efficacy and potential side effects of DBT for management of postmenopausal osteoporosis.

## Ethics Statement

The present experiment was conducted under the animal license issued by Department of Health, Hong Kong Special Administrative Region Government, and The Hong Kong Polytechnic University Animal Subjects Ethics Sub-committee (Animal License No. 13-104; ASESC Case: 12/11). Six-month-old female Sprague-Dawley rats weighing 230 ± 20 g were purchased from The Chinese University of Hong Kong and housed in Centralized Animal Facilities (CAF) of The Hong Kong Polytechnic University on a 12 h light/dark cycle. Water and food were available *ad libitum*. After 1 week of acclimatization, all the rats were given bilateral ovariectomy or sham operation under anesthesia with ketamine and xylazine. Upon recovery for 2 weeks, the OVX rats were randomly divided into seven treatment groups and orally administrated with vehicle, 17β-estradiol (2 mg/kg.day) as positive control, tamoxifen (1 mg/kg.day), raloxifene (3 mg/kg.day), DBT (3 g/kg.day) as well as combinations of DBT and either tamoxifen or raloxifene for 12 consecutive weeks. During the recovery period, they are inspected each day in order to check the condition of the wound and to see if there is any sign of pain, e.g., reduced appetite, inactive. Upon treatment, rats were sacrificed under anesthesia with ketamine/xylazine and samples like urine, blood, uterus, bone, and spine were collected for further measurement.

## Author Contributions

L-PZ planned and performed experiments and statistical analysis, and wrote the manuscript. K-YW, H-TY, X-LD, and H-HX helped in planning and performing animal experiments. KT involved in the design of animal and cell culture experiments and provided the DBT extract and AG helped with the chemical analysis of DBT extract. M-SW conceived and supervised the experiments and finalized the manuscript. All authors reviewed the manuscript.

## Conflict of Interest Statement

The authors declare that the research was conducted in the absence of any commercial or financial relationships that could be construed as a potential conflict of interest.

## References

[B1] Ahmet-Camcioglu N. Okman-Kilic T. Durmus-Altun G. Ekuklu G. Kucuk M. (2009). Effects of strontium ranelate, raloxifene and misoprostol on bone mineral density in ovariectomized rats. *Eur. J. Obstet. Gynecol. Reprod. Biol.* 147 192–194. 10.1016/j.ejogrb.2009.09.001 19796864

[B2] Bedell S. Nachtigall M. Naftolin F. (2014). The pros and cons of plant estrogens for menopause. *J. Steroid Biochem. Mol. Biol.* 139 225–236. 10.1016/j.jsbmb.2012.12.004 23270754

[B3] Borrelli F. Ernst E. (2010). Alternative and complementary therapies for the menopause. *Maturitas* 66 333–343. 10.1016/j.maturitas.2010.05.010 20580501

[B4] Callaci J. J. Himes R. Lauing K. Wezeman F. H. Brownson K. (2009). Binge alcohol-induced bone damage is accompanied by differential expression of bone remodeling-related genes in rat vertebral bone. *Calcif. Tissue Int.* 84 474–484. 10.1007/s00223-009-9240-z 19330277 PMC2693714

[B5] Canpolat S. Tug N. Seyran A. D. Kumru S. Yilmaz B. (2010). Effects of raloxifene and estradiol on bone turnover parameters in intact and ovariectomized rats. *J. Physiol. Biochem.* 66 23–28. 10.1007/s13105-010-0008-8 20428990

[B6] Choi R. C. Gao Q. T. Cheung A. W. Zhu J. T. Lau F. T. Li J. (2011). A Chinese herbal decoction, danggui buxue tang, stimulates proliferation, differentiation and gene expression of cultured osteosarcoma cells: genomic approach to reveal specific gene activation. *Evid. Based Complement. Alternat. Med.* 2011:307548. 10.1093/ecam/nen085 19131392 PMC3136360

[B7] Cooke A. L. Metge C. Lix L. Prior H. J. Leslie W. D. (2008). Tamoxifen use and osteoporotic fracture risk: a population-based analysis. *J. Clin. Oncol.* 26 5227–5232. 10.1200/Jco.2007.15.7123 18838712

[B8] Davison S. Davis S. R. (2003). Hormone replacement therapy: current controversies. *Clin. Endocrinol.* 58 249–261. 10.1046/j.1365-2265.2003.01774.x 12608928

[B9] de Villiers T. J. Gass M. L. Haines C. J. Hall J. E. Lobo R. A. Pierroz D. D. (2013). Global consensus statement on menopausal hormone therapy. *Climacteric* 16 203–204. 10.3109/13697137.2013.771520 23488524

[B10] Dong T. T. Zhao K. J. Gao Q. T. Ji Z. N. Zhu T. T. Li J. (2006). Chemical and biological assessment of a Chinese herbal decoction containing radix Astragali and radix Angelicae Sinensis: determination of drug ratio in having optimized properties. *J. Agric. Food Chem.* 54 2767–2774. 10.1021/jf053163l 16569074

[B11] Ettinger B. Black D. M. Mitlak B. H. Knickerbocker R. K. Nickelsen T. Genant H. K. (1999). Reduction of vertebral fracture risk in postmenopausal women with osteoporosis treated with raloxifene: results from a 3-year randomized clinical trial. Multiple outcomes of raloxifene evaluation (MORE) investigators. *JAMA* 282 637–645. 10.1001/jama.282.7.637 10517716

[B12] Gao Q. T. Choi R. C. Cheung A. W. Zhu J. T. Li J. Chu G. K. (2007). Danggui buxue tang–a Chinese herbal decoction activates the phosphorylations of extracellular signal-regulated kinase and estrogen receptor alpha in cultured MCF-7 cells. *FEBS Lett.* 581 233–240. 10.1016/j.febsiet.2006.12.018 17187784

[B13] Garcia-Martin A. Reyes-Garcia R. Garcia-Castro J. M. Rozas-Moreno P. Escobar-Jimenez F. Munoz-Torres M. (2012). Role of serum FSH measurement on bone resorption in postmenopausal women. *Endocrine* 41 302–308. 10.1007/s12020-011-9541-7 21964645

[B14] Gong A. G. Lau K. M. Xu M. L. Lin H. Q. Dong T. T. Zheng K. Y. (2016). The estrogenic properties of danggui buxue tang, a Chinese herbal decoction, are triggered predominantly by calycosin in MCF-7 cells. *J. Ethnopharmacol.* 189 81–89. 10.1016/j.jep.2016.05.035 27196297

[B15] Goss P. E. Qi S. Hu H. (2009). Comparing the effects of atamestane, toremifene and tamoxifen alone and in combination, on bone, serum lipids and uterus in ovariectomized rats. *J. Steroid Biochem. Mol. Biol.* 113 233–240. 10.1016/j.jsbmb.2009.01.005 19429427

[B16] Goss P. E. Strasser-Weippl K. Qi S. Hu H. (2007). Effects of liarozole fumarate (R85246) in combination with tamoxifen on N-methyl-N-nitrosourea (MNU)-induced mammary carcinoma and uterus in the rat model. *BMC Cancer* 7:26. 10.1186/1471-2407-7-26 17266767 PMC1796889

[B17] Khalid A. B. Krum S. A. (2016). Estrogen receptors alpha and beta in bone. *Bone* 87 130–135. 10.1016/j.bone.2016.03.016 27072516 PMC5336249

[B18] Komm B. S. Mirkin S. (2014). An overview of current and emerging SERMs. *J. Steroid Biochem. Mol. Biol.* 143 207–222. 10.1016/j.jsbmb.2014.03.003 24667357

[B19] Lau W. S. Chen W. F. Chan R. Y. Guo D. A. Wong M. S. (2009). Mitogen-activated protein kinase (MAPK) pathway mediates the oestrogen-like activities of ginsenoside Rg1 in human breast cancer (MCF-7) cells. *Br. J. Pharmacol.* 156 1136–1146. 10.1111/j.1476-5381.2009.00123.x 19298253 PMC2697688

[B20] Lindsay R. (2004). Hormones and bone health in postmenopausal women. *Endocrine* 24 223–230. 10.1385/ENDO:24:3:223 15542889

[B21] Maricic M. Gluck O. (2002). Review of raloxifene and its clinical applications in osteoporosis. *Expert Opin. Pharmacother.* 3 767–775. 10.1517/14656566.3.6.767 12036416

[B22] Nath A. Sitruk-Ware R. (2009). Pharmacology and clinical applications of selective estrogen receptor modulators. *Climacteric* 12 188–205. 10.1080/13697130802657896 19387883

[B23] Nowacka-Cieciura E. Sadowska A. Pacholczyk M. Chmura A. Tronina O. Durlik M. (2016). Bone mineral density and bone turnover markers under bisphosphonate therapy used in the first year after liver transplantation. *Ann. Transplant.* 21 241–249. 10.12659/Aot.895413 27112626

[B24] Pang W. Y. Wang X. L. Mok S. K. Lai W. P. Chow H. K. Leung P. C. (2010). Naringin improves bone properties in ovariectomized mice and exerts oestrogen-like activities in rat osteoblast-like (UMR-106) cells. *Br. J. Pharmacol.* 159 1693–1703. 10.1111/j.1476-5381.2010.00664.x 20397301 PMC2925492

[B25] Parthan A. Kruse M. Yurgin N. Huang J. Viswanathan H. N. Taylor D. (2013). Cost effectiveness of denosumab versus oral bisphosphonates for postmenopausal osteoporosis in the US. *Appl. Health Econ. Health Policy* 11 485–497. 10.1007/s40258-013-0047-8 23868102

[B26] Recker R. R. Kimmel D. B. Dempster D. Weinstein R. S. Wronski T. J. Burr D. B. (2011). Issues in modern bone histomorphometry. *Bone* 49 955–964. 10.1016/j.bone.2011.07.017 21810491 PMC3274956

[B27] Sowers M. R. Greendale G. A. Bondarenko I. Finkelstein J. S. Cauley J. A. Neer R. M. (2003). Endogenous hormones and bone turnover markers in pre- and perimenopausal women: SWAN. *Osteoporos. Int.* 14 191–197. 10.1007/s00198-002-1329-4 12730778

[B28] Stein E. M. Kepley A. Walker M. Nickolas T. L. Nishiyama K. Zhou B. (2014). Skeletal structure in postmenopausal women with osteopenia and fractures is characterized by abnormal trabecular plates and cortical thinning. *J. Bone Miner. Res.* 29 1101–1109. 10.1002/jbmr.2144 24877245 PMC4084559

[B29] Sun L. Peng Y. Sharrow A. C. Iqbal J. Zhang Z. Papachristou D. J. (2006). FSH directly regulates bone mass. *Cell* 125 247–260. 10.1016/j.cell.2006.01.051 16630814

[B30] Taylor H. S. (2009). Designing the ideal selective estrogen receptor modulator–an achievable goal? *Menopause* 16 609–615. 10.1097/gme.0b013e3181906fa3 19182697 PMC3107842

[B31] Tzeng H. E. Muo C. H. Chen H. T. Hwang W. L. Hsu H. C. Tsai C. H. (2015). Tamoxifen use reduces the risk of osteoporotic fractures in women with breast cancer in Asia: a nationwide population-based cohort study. *BMC Musculoskelet. Disord.* 16:123. 10.1186/s12891-015-0580-8 25989902 PMC4438570

[B32] Wang C. C. Cheng K. F. Lo W. M. Law C. Li L. Leung P. C. (2013). A randomized, double-blind, multiple-dose escalation study of a Chinese herbal medicine preparation (Dang Gui Buxue Tang) for moderate to severe menopausal symptoms and quality of life in postmenopausal women. *Menopause* 20 223–231. 10.1097/gme.0b013e318267f64e 22990757

[B33] Weinreb M. Shinar D. Rodan G. A. (1990). Different pattern of alkaline phosphatase, osteopontin, and osteocalcin expression in developing rat bone visualized by in situ hybridization. *J. Bone Miner. Res.* 5 831–842. 10.1002/jbmr.5650050806 2239367

[B34] Wood C. E. Register T. C. Cline J. M. (2007). Soy isoflavonoid effects on endogenous estrogen metabolism in postmenopausal female monkeys. *Carcinogenesis* 28 801–808. 10.1093/carcin/bgl163 17032659

[B35] Yeh J. K. Chen M. M. Aloia J. F. (1996). Ovariectomy-induced high turnover in cortical bone is dependent on pituitary hormone in rats. *Bone* 18 443–450. 10.1007/s13105-010-0008-8 8739902

[B36] Yeh J. K. Chen M. M. Aloia J. F. (1997). Effects of 17 beta-estradiol administration on cortical and cancellous bone of ovariectomized rats with and without hypophysectomy. *Bone* 20 413–420. 10.1016/S8756-3282(97)00027-6 9145238

[B37] Zebaze R. M. Ghasem-Zadeh A. Bohte A. Iuliano-Burns S. Mirams M. Price R. I. (2010). Intracortical remodelling and porosity in the distal radius and post-mortem femurs of women: a cross-sectional study. *Lancet* 375 1729–1736. 10.1016/S0140-6736(10)60320-0 20472174

[B38] Zhang Y. Chen W. F. Lai W. P. Wong M. S. (2008). Soy isoflavones and their bone protective effects. *Inflammopharmacology* 16 213–215. 10.1007/s10787-008-8018-7 18815742

[B39] Zheng K. Y. Choi R. C. Guo A. J. Bi C. W. Zhu K. Y. Du C. Y. (2012). The membrane permeability of Astragali radix-derived formononetin and calycosin is increased by Angelicae Sinensis radix in Caco-2 cells: a synergistic action of an ancient herbal decoction Danggui buxue tang. *J. Pharm. Biomed. Anal.* 70 671–679. 10.1016/j.jpba.2012.05.018 22704738

[B40] Zierau O. Zheng K. Y. Z. Papke A. Dong T. T. X. Tsim K. W. K. Vollmer G. (2014). Functions of Danggui buxue tang, a Chinese herbal decoction containing Astragali radix and Angelicae Sinensis radix, in uterus and liver are both estrogen receptor-dependent and -independent. *Evid. Based Complement. Alternat. Med.* 2014:438531. 10.1155/2014/438531 25214874 PMC4156991

